# Relation between respiratory variations in pulse oximetry plethysmographic waveform amplitude and arterial pulse pressure in ventilated patients

**DOI:** 10.1186/cc3799

**Published:** 2005-08-23

**Authors:** Maxime Cannesson, Cyril Besnard, Pierre G Durand, Julien Bohé, Didier Jacques

**Affiliations:** 1 Anesthesiology and Critical Care Fellow, Service de Réanimation Médicale, Centre Hospitalier Lyon Sud, Pierre Bénite, France; 2 Intensivist, Service de Réanimation Médicale, Centre Hospitalier Lyon Sud, Pierre Bénite, France; 3 Intensivist, Service de Réanimation Médicale, Centre Hospitalier Lyon Sud, Pierre Bénite, France

## Abstract

**Introduction:**

Respiratory variation in arterial pulse pressure is a reliable predictor of fluid responsiveness in mechanically ventilated patients with circulatory failure. The main limitation of this method is that it requires an invasive arterial catheter. Both arterial and pulse oximetry plethysmographic waveforms depend on stroke volume. We conducted a prospective study to evaluate the relationship between respiratory variation in arterial pulse pressure and respiratory variation in pulse oximetry plethysmographic (POP) waveform amplitude.

**Method:**

This prospective clinical investigation was conducted in 22 mechanically ventilated patients. Respiratory variation in arterial pulse pressure and respiratory variation in POP waveform amplitude were recorded simultaneously in a beat-to-beat evaluation, and were compared using a Spearman correlation test and a Bland–Altman analysis.

**Results:**

There was a strong correlation (r^2 ^= 0.83; *P *< 0.001) and a good agreement (bias = 0.8 ± 3.5%) between respiratory variation in arterial pulse pressure and respiratory variation in POP waveform amplitude. A respiratory variation in POP waveform amplitude value above 15% allowed discrimination between patients with respiratory variation in arterial pulse pressure above 13% and those with variation of 13% or less (positive predictive value 100%).

**Conclusion:**

Respiratory variation in arterial pulse pressure above 13% can be accurately predicted by a respiratory variation in POP waveform amplitude above 15%. This index has potential applications in patients who are not instrumented with an intra-arterial catheter.

## Introduction

Initial therapy in patients with sepsis-induced circulatory failure is volume expansion. However, fluid therapy is not always efficient and does not always increase stroke volume. Furthermore, fluid therapy carries major risks for complications such as volume overload, systemic and pulmonary oedema, and increased tissue hypoxia [1]. To avoid the potential deleterious effects of volume expansion, reliable predictors of fluid responsiveness are needed. In mechanically ventilated patients, respiratory variations in systemic systolic pressure (ΔPs) have been proposed to be a good indicator of fluid responsiveness [2,3]. Indeed, fluid responsiveness was found to be proportional to ΔPs. More recently, respiratory variations in systemic pulse pressure (ΔPP) were shown to be even more predictive of fluid responsiveness [4]. ΔPP above 13% could predict an increase in cardiac index of 15% or more after infusion of 500 ml colloids with positive and negative predictive values of 94% and 96%, respectively. One of the limitations of this method is that it requires an intra-arterial catheter, and catheter-related sepsis and ischaemia are well known complications of the use of such devices [5,6]. Furthermore, most patients are not equipped with such a device when the circulatory failure manifests.

Pulse oximeters are widely used in intensive care units. The pulse oximetry plethysmographic (POP) waveform depends on arterial pulsatility. Respiratory variations in POP waveform peaks are correlated with ΔPs [7] in the setting of mechanical ventilation. However, respiratory variations in POP waveform amplitude (ΔPOP) have not been evaluated. Thus, we tested the hypothesis that ΔPOP and ΔPP are correlated in mechanically ventilated critically ill patients.

## Materials and methods

The protocol used in the present study was part of our routine clinical practice, and ethical approval was given by the institutional review board (Comité Consultatif de Protection des Personnes dans la Recherche Biomédicale Lyon B) of our institution (Hospices Civils de Lyon, France).

### Patients

Twenty-two deeply sedated patients (14 men and 8 women) receiving mechanical ventilation were studied. Their age (mean ± standard deviation) was 64 ± 11 years (range 41–85 years). Inclusion criteria were as follows: instrumentation with an indwelling radial arterial catheter, according to the attending physician; haemodynamic stability, defined as a variation in heart rate and blood pressure of less than 10% over the 15 min preceding the period of evaluation; and pulse oximetry monitored using a pulse oximeter (M1190A; Philips, Suresnes, France) attached to the index or middle finger. Exclusion criteria were cardiac arrhythmia and low POP signal. POP waveform quality was considered suitable when POP amplitude was superior to the signal quality index displayed by the monitor.

### Haemodynamic measurements

Patients were studied in supine position. The arterial pressure transducer was set at mid-axillary level for zero pressure. When available, transthoracic echocardiography was performed to assess left ventricular function. Left ventricular systolic dysfunction was defined as left ventricular ejection fraction below 40%.

### Respiratory variations in arterial pulse pressure analysis

Pulse pressure (PP) was calculated at the bedside using a standard monitor (Monitor M1165A; Philips) as the difference between systolic and diastolic arterial pressures. Maximal PP (PPmax) and minimal PP (PPmin) values were determined over the same respiratory cycle. To assess ΔPP, the percentage change in PP was calculated (as described by Michard and coworkers [4]): ΔPP (%) = 100 × ([PPmax - PPmin]/ [(PPmax + PPmin)/2]). The measurements were repeated on three consecutive respiratory cycles and averaged for statistical analysis.

### Respiratory variations in POP waveform amplitude analysis

**Figure 1:**
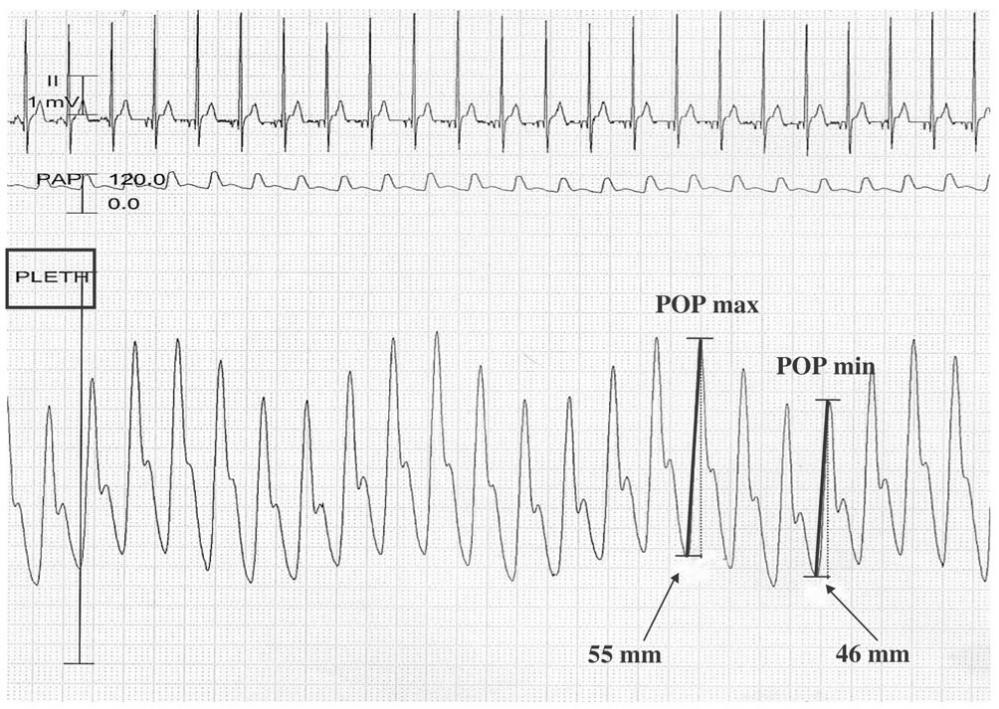
Pulse oximetry plethysmographic waveform analysis. Shown is pulse oximetry plethysmographic (POP) waveform (PLETH) analysis in one illustrative patient. Beat-to-beat measurement of POP waveform amplitude allowed determination of maximal POP (POPmax) and minimal POP (POPmin) over a single respiratory cycle.

A pulse oximeter was attached to the index or middle finger of either right or left hand. POP waveforms were recorded using a monitor (M3150A; Philips). The plethysmographic gain factor was held constant throughout the procedure, which was possible because the bedside monitor allows one to choose between manual and automatic gain control. POP waveform amplitude was measured on a beat-to-beat basis as the vertical distance between peak and preceding valley trough in the waveform and was expressed in millimeters (Fig. 1). Maximal POP (POPmax) and minimal POP (POPmin) were determined over the same respiratory cycle. ΔPOP were calculated using a formula similar to that for ΔPP: ΔPOP (%) = 100 × ([POPmax - POPmin]/ [(POPmax + POPmin)/2]). ΔPOP was evaluated on three consecutive respiratory cycles simultaneously with ΔPP measurements. Measurements were then averaged for statistical analysis.

### Respiratory parameters

All patients received mechanical ventilation in volume controlled mode with a tidal volume of 8 ± 2 ml/kg and an inspiratory/expiratory ratio of one-third to one-half. Positive end-expiratory pressure was set at 5 ± 4 cmH_2_O.

### Statistical analysis

Linear correlation between ΔPP and ΔPOP was tested using the Spearman rank method. ΔPP and ΔPOP were compared using Bland–Altman analyses [8]. Data are presented as mean ± standard deviation. A receiver operating characteristic curve was generated for ΔPOP, varying the discriminating threshold of this parameter to determine the ability of ΔPOP to discriminate between patients with a ΔPP above 13% and those with a ΔPP of 13% or less. *P *< 0.05 was considered statistically significant. Statistical analysis was performed using Statview 5.0 software (SAS Institute Inc., Cary, NC, USA).

## Results

Among the 22 patients studied, acute circulatory failure (defined as a systolic blood pressure <90 mmHg or need for vasopressive drugs) was present in 14 patients (12 received vasopressor support and two had severe hypotension). Sepsis (*n *= 7) and bleeding (*n *= 3) were the main causes. Other patients had isolated acute respiratory failure.

Echocardiography was performed in 19 patients (86%), revealing left ventricular systolic dysfunction in five patients. Other demographic, haemodynamic and ventilatory parameters are presented in Table 1.

**Figure 2:**
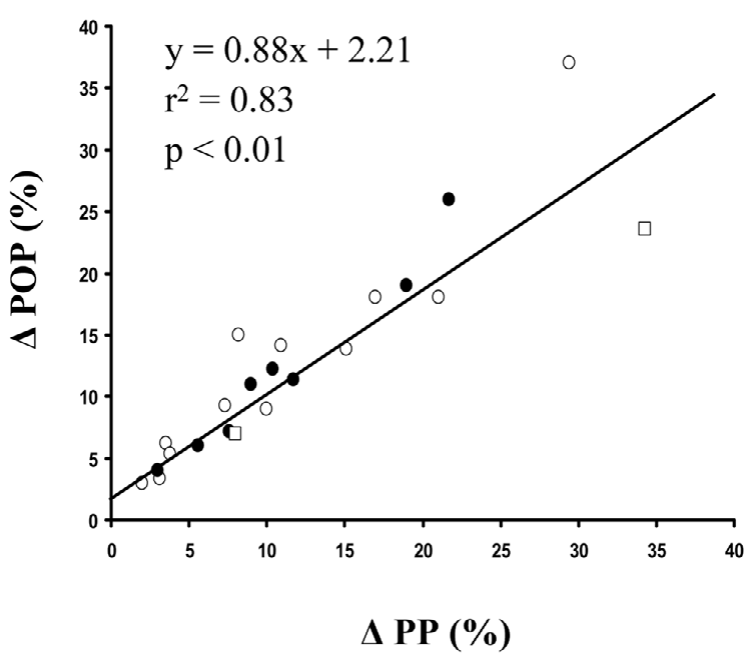
Relationship between ΔPOP and ΔPP. Empty circles indicate patients receiving vasopressor support, and empty squares indicate patients with severe hypotension. ΔPOP, respiratory variations in POP waveform amplitude; ΔPP, respiratory variations in systemic pulse pressure.

**Figure 3:**
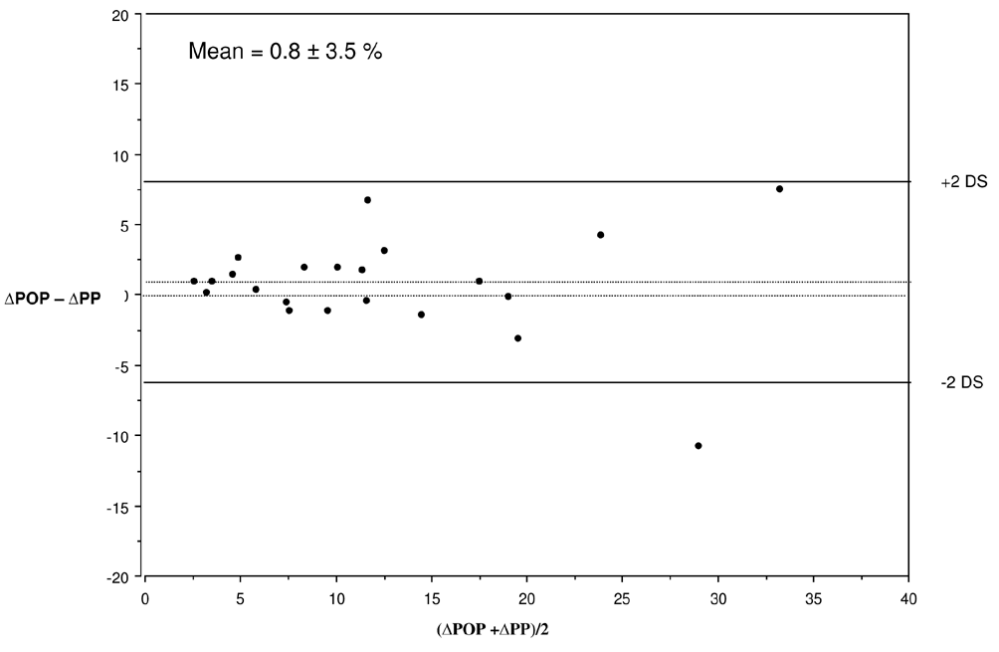
Bias and precision of ΔPP estimated from ΔPOP (Bland–Altman analysis). ΔPOP, respiratory variations in POP waveform amplitude; ΔPP, respiratory variations in systemic pulse pressure.

**Figure 4:**
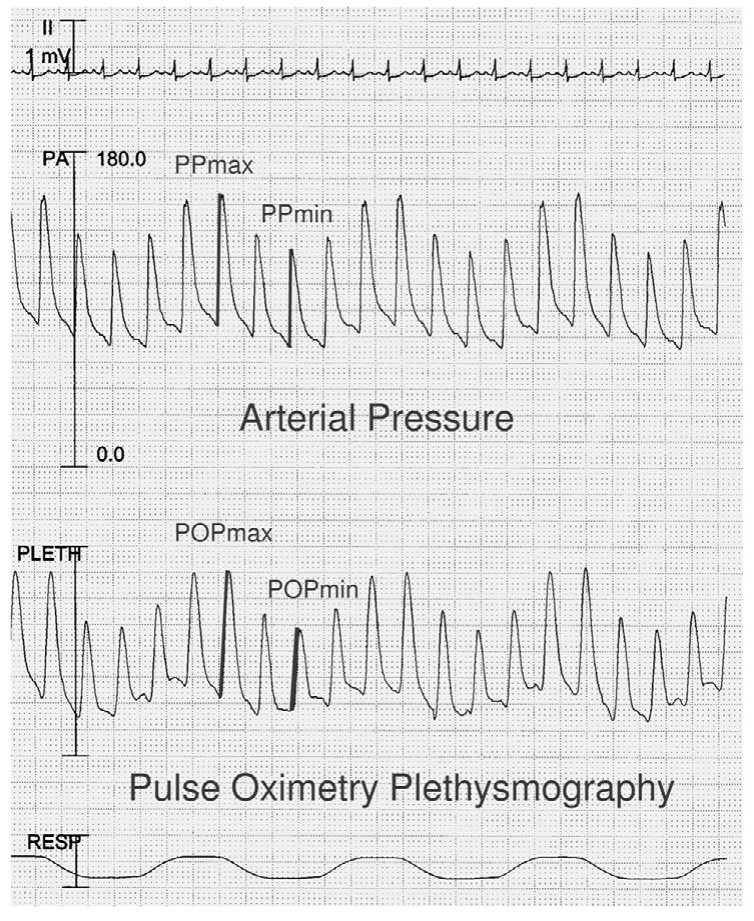
Comparison between invasive arterial pressure and pulse oxymetry plethysmography recordings. Simultaneous recording of electrocardiographic lead (II), systemic arterial pressure (PA), pulse oximetry plethysmography (PLETH) and respiratory signal (RESP) in one illustrative patient. POP, pulse oximetry plethysmographic; PP, pulse pressure.

In the patients overall, there was a strong correlation between ΔPP and ΔPOP (r^2 ^= 0.83; *P *< 0.001), as shown in Fig. 2. This correlation remained significant in the subgroup of 14 patients with acute circulatory failure (r^2 ^= 0.89; *P *< 0.001). Using Bland–Altman analysis (Fig. 3), there was a weak bias and relatively good precision between the two methods (0.8 ± 3.5%). Figure 4 shows an example of simultaneous recording of arterial pressure and pulse oxymetry plethysmography in a patient with gastric bleeding. The threshold ΔPOP value of 15% permitted discrimination between patients with a ΔPP above 13% and those with a ΔPP of 13% or less with a sensitivity of 87%, a specificity of 100%, a positive predictive value of 100% and a negative predictive value of 94%.

## Discussion

ΔPP is an invasive but accurate indicator of fluid responsiveness in mechanically ventilated patients with acute circulatory failure [4]. This study demonstrates a close relationship between the noninvasively measured parameter ΔPOP and ΔPP. A patient with a ΔPOP value above 15% was highly likely to have a ΔPP value of above 13% (positive predictive value 100%). In contrast, if ΔPOP was below 15% then the patient was unlikely to have a ΔPP value of more than 13% (negative predictive value 94%). However, it must be recalled that 13% is not a universal cutoff value and that many studies focusing on fluid responsiveness and ΔPP have found values ranging from 11% to 13%; we used 13% as a reference because it was the first cutoff value to be reported [9,10]. The pulse oximeter is a standard noninvasive monitor in intensive care units and operating rooms, and is used to monitor arterial oxygen saturation. Our data suggest that the pulse oximeter could also be used to assess fluid responsiveness, but further studies with volume expansion are needed to address this and to determine the optimal ΔPOP threshold.

Many indices have been proposed for monitoring fluid therapy in patients with acute circulatory failure induced by hypovolaemia or severe sepsis. Right or left ventricular filling pressures and cardiac volume measurements have several limitations [11]. In sedated and mechanically ventilated patients, respiratory variations in arterial pressure have been studied for more than 20 years [2,3,12,13], showing that the degree of hypovolaemia correlates closely with ΔPs. Indeed, inspiratory right ventricular stroke volume decrease is proportional to the degree of hypovolaemia and is transmitted to the left heart after two or three beats. Thus, left ventricular stroke volume and then arterial pressure decrease during expiration. More recently, ΔPP were shown to be slightly more predictive of fluid responsiveness than were ΔPs [4]. Indeed, ΔPs depend not only on respiration induced changes in stroke volume but also on respiration induced changes in intrathoracic pressure, which are transmitted to both diastolic and systolic components of blood pressure. On the other hand, PP variations do not depend on intrathoracic pressure variations and therefore are more related to stroke volume variations than variations in systolic pressure [4].

Pulse oximeters display a signal proportional to light absorption between the nail and the anterior face of the finger. Light absorption increases with the amount of haemoglobin present in the fingertip. Thus, the POP waveform depends on the arterial pulse [14]. Previous studies have shown a correlation between respiratory variations in POP waveform peaks and arterial systolic pressure [7,15], demonstrating that decreased preload resulted in waveform variation of the plethysmographic signal similar to the variation observed in the arterial waveform. However, like systolic pressure, POP waveform peaks also depend on transmission of intrathoracic pressure [14]. Hence, POP waveform amplitude analysis should be more accurate. To the best of our knowledge, a relationship between ΔPOP and ΔPP has not yet been reported. PP and POP waveform amplitude are related to stroke volume and vascular tone [14]. Vascular tone is considered unchanged between inspiration and expiration, and so respiratory variations in POP waveform amplitude mainly reflect respiratory changes in left ventricular stroke volume.

Because pulse oximeters are already widely available in intensive care units and operating rooms, they may represent a noninvasive and simple means with which to predict fluid responsiveness in patients with circulatory failure, especially if they are not instrumented with an arterial catheter. Because most patients with shock have arterial catheters, POP waveform analysis could be utilized in patients not routinely monitored with such catheters. Applications include detection and assessment of unexpected circulatory failure in patients undergoing surgery, and preliminary evaluation of patients admitted for shock to intensive care units.

### Study limitations

Only 14 out of 22 patients in this series presented with circulatory failure. Seven of them seemed fluid dependent, according to their ΔPP value. It must be emphasized that our study focused on the relationship between respiratory variations in both POP waveform amplitude and arterial PP. Although there was good agreement between ΔPOP and ΔPP, the precision was quite low (3.5%), especially for the highest values (Figs 2 and 3). This means that ΔPP cannot be accurately inferred from ΔPOP. Consequently, further experiments are required to study the relationship between ΔPOP and fluid responsiveness and to determine the optimal threshold value for ΔPOP in patients with acute circulatory failure. Such studies should also focus on the evolution of ΔPOP after volume expansion. Moreover, we chose 13% as a cutoff value for ΔPP because this was the first value to be reported and because most of the studies focusing on this topic found similar values. However, even though a ΔPOP above 15% appears to be a strong predictor of a ΔPP above 13%, it must be emphasized that a prospective study using volume expansion is needed to determine the optimal ΔPOP threshold. Nevertheless, our study is the first to demonstrate a strong relationship between ΔPOP and ΔPP and our findings should be considered to provide a primary hypothesis for such experiments.

As is the case with ΔPP, ΔPOP cannot be assessed in the case of cardiac arrhythmia or in patients who trigger the respirator. Also, the POP signal can be unstable, depending on finger perfusion. Therefore, a stable and satisfactory signal is a prerequisite for assessing ΔPOP. Next, commercially available pulse oximeters use automated algorithms to display a stable signal by adjusting gain continuously. Automatic gain must therefore be disabled to allow respiratory variations to emerge. Finally, the POP waveform is a scaleless curve. Thus, only relative changes in POP waveform amplitude (ΔPOP) can be used to assess volume status, and not absolute values. Because currently there are no commercial monitors that display ΔPOP values, the POP waveform may be used by the clinician by visual inspection alone to assess volume status.

## Conclusion

The results of the present study show that there is a strong correlation and a relatively good agreement between ΔPOP and ΔPP. Moreover, a ΔPOP value of more than 15% accurately discriminated patients with a ΔPP above 13% from those with a ΔPP of 13 or less. Therefore, ΔPOP has potential in assessing ΔPP in patients who are not instrumented with an intra-arterial catheter.

## Key messages

• 	ΔPOP and ΔPP are strongly correlated.

• 	A ΔPOP value above 15% accurately discriminates patients with a ΔPP above 13% from those with a ΔPP of 13 or less.

• 	ΔPOP has potential for clinical application in the assessment of fluid responsiveness, but further studies are required to address this.

## Abbreviations

ΔPOP = respiratory variations in POP waveform amplitude; ΔPP = respiratory variations in systemic pulse pressure; ΔPs = respiratory variations in systemic systolic pressure; POP = pulse oximetry plethysmographic; PP = pulse pressure.

## Competing interests

The author(s) declare that they have no competing interests.

## Authors' contributions

MC conceived the study, analyzed the curves, performed the statistical analysis and drafted the manuscript. CB and PGD collected the data and helped to draft the manuscript. JB participated in the design of the study. DJ conceived the study, participated in its design and coordination, and helped to draft the manuscript. All authors read and approved the final manuscript.

## Figures and Tables

**Table 1:** Demographic data and baseline values for haemodynamic, plethysmographic and respiratory parameters

Parameter	Value (mean ± SD)	Range
Demography		
Age (year)	64 ± 11	41–85
Height (cm)	166 ± 8	155–180
Weight (kg)	65 ± 12	106–17
Arterial blood pressure and heart rate		
Systolic blood pressure (mmHg)	116 ± 20	78–146
Diastolic blood pressure (mmHg)	57 ± 14	39–89
Mean arterial pressure (mmHg)	75 ± 15	54–105
Heart rate (beats/min)	97 ± 19	66–133
PPmax (mmHg)	66 ± 15	39–95
PPmin (mmHg)	60 ± 16	29–90
ΔPP (%)	12 ± 8	2–34
Pulse oximetry plethysmography		
POPmax (mm)	52 ± 4	43–61
POPmin (mm)	46 ± 3	37–51
ΔPOP (%)	12 ± 8	3–37
SpO_2 _(%)	96 ± 3	90–100
Respiratory parameters		
PaO_2_/FiO_2 _(mmHg)	233 ± 99	67–440
Vt (ml/kg)	8 ± 2	5–13
Respiratory rate (breaths/min)	19 ± 5	12–30
PEEP (cm H_2_O)	5 ± 4	0–15

ΔPOP, respiratory variations in pulse oximetry plethysmographic waveform amplitude; ΔPP, respiratory variations in pulse pressure; PaO_2_/FiO_2_, ratio of arterial oxygen tension to fractional inspired oxygen; PEEP, positive end-expiratory pressure; SpO_2_, pulse oximeter oxygen saturation; Vt, tidal volume.

## References

[B1] Anonymous (1999). Practice parameters for hemodynamic support of sepsis in adult patients in sepsis. Task Force of the American College of Critical Care Medicine, Society of Critical Care Medicine. Crit Care Med.

[B2] Coriat P, Vrillon M, Perel A, Baron JF, Le Bret F, Saada M, Viars P (1994). A comparison of systolic blood pressure variations and echocardiographic estimates of end-diastolic left ventricular size in patients after aortic surgery. Anesth Analg.

[B3] Tavernier B, Makhotine O, Lebuffe G, Dupont J, Scherpereel P (1998). Systolic pressure variation as a guide to fluid therapy in patients with sepsis-induced hypotension. Anesthesiology.

[B4] Michard F, Boussat S, Chemla D, Anguel N, Mercat A, Lecarpentier Y, Richard C, Pinsky MR, Teboul JL (2000). Relation between respiratory changes in arterial pulse pressure and fluid responsiveness in septic patients with acute circulatory failure. Am J Respir Crit Care Med.

[B5] Bedford RF, Wollman H (1973). Complications of percutaneous radial-artery cannulation: an objective prospective study in man. Anesthesiology.

[B6] Jones RM, Hill AB, Nahrwold ML, Bolles RE (1981). The effect of method of radial artery cannulation on postcannulation blood flow and thrombus formation. Anesthesiology.

[B7] Shamir M, Eidelman LA, Floman Y, Kaplan L, Pizov R (1999). Pulse oximetry plethysmographic waveform during changes in blood volume. Br J Anaesth.

[B8] Bland JM, Altman DG (1986). Statistical methods for assessing agreement between two methods of clinical measurement. Lancet.

[B9] Vieillard-Baron A, Chergui K, Rabiller A, Peyrouset O, Page B, Beauchet A, Jardin F (2004). Superior vena caval collapsibility as a gauge of volume status in ventilated septic patients. Intensive Care Med.

[B10] Kramer A, Zygun D, Hawes H, Easton P, Ferland A (2004). Pulse pressure variation predicts fluid responsiveness following coronary artery bypass surgery. Chest.

[B11] Michard F, Teboul JLL (2002). Predicting fluid responsiveness in ICU patients: a critical analysis of the evidence. Chest.

[B12] Coyle JP, Teplick RS, Long MC, Davison JK (1983). Respiratory variations in systemic arterial pressure as an indicator of volume status. Anesthesiology.

[B13] Perel A, Pizov R, Cotev S (1987). Systolic blood pressure variation is a sensitive indicator of hypovolemia in ventilated dogs subjected to graded hemorrhage. Anesthesiology.

[B14] Dorlas JC, Nijboer JA (1985). Photo-electric plethysmography as a monitoring device in anaesthesia. Application and interpretation. Br J Anaesth.

[B15] Partridge BL (1987). Use of pulse oximetry as a non invasive indicator of intravascular volume status. J Clin Monit.

